# Thrombocytopenia and PT-INR in patients with anorexia nervosa and severe liver dysfunction

**DOI:** 10.1186/s13030-023-00269-2

**Published:** 2023-03-08

**Authors:** Ken Kurisu, Kaoruko Sato, Mikiko Matsuoka, Makoto Otani, Kazuhiro Yoshiuchi

**Affiliations:** Department of Stress Sciences and Psychosomatic Medicine, Graduate School of Medicine, The University of Tokyo, 7-3-1, Hongo, Bunkyo-Ku, Tokyo, 113-8655 Japan

**Keywords:** Eating disorders, Anorexia nervosa, Liver dysfunction, Platelet, Thrombocytopenia, PT-INR, Thrombopoietin

## Abstract

**Background:**

We previously reported a case that led us to hypothesize that decreased production of thrombopoietin (TPO) leads to thrombocytopenia in patients with anorexia nervosa (AN) with severe liver dysfunction and that prolonged prothrombin time-international normalized ratio (PT-INR) predicts thrombocytopenia in such cases. To validate this hypothesis, we report another case in which TPO levels were measured. In addition, we examined the association between prolonged PT-INR and thrombocytopenia in such patients.

**Main body:**

Similar to the previously reported patient, a patient with AN with severe liver dysfunction showed that TPO levels increased after improvements in liver enzyme levels and PT-INR, followed by recovery of platelet count. In addition, a retrospective study was also conducted to review patients with AN whose liver enzyme levels were > 3 × the upper limit of normal (aspartate aminotransferase > 120 U/L or alanine aminotransferase > 135 U/L). The study included 58 patients and showed a correlation coefficient of -0.486 (95% confidence interval [CI], -0.661 to -0.260; *P* < 0.001) between maximum PT-INR and minimum platelet count. These patients showed higher PT-INR (β, 0.07; 95% CI, 0.02 to 0.13; *P* = 0.005) and lower platelet count (β, -5.49; 95% CI, -7.47 to -3.52; *P* < 0.001) than the 58 matched control patients without severe liver dysfunction, even after adjusting for body mass index.

**Conclusions:**

In patients with AN with severe liver dysfunction, prolongation of PT-INR could predict thrombocytopenia, which may be mediated by decreased TPO production due to decreased hepatic synthetic function.

## Background

Liver dysfunction is a frequent complication of anorexia nervosa (AN), caused by starvation-induced autophagy, liver ischemia, and refeeding syndrome [1–4]. Patients with AN often present with thrombocytopenia [5–7], reportedly caused by bone marrow hypoplasia [8]. However, the association between liver dysfunction and thrombocytopenia in AN has not been fully explored.

Thrombopoietin (TPO) is a regulatory factor in platelet production and is mainly synthesized in the liver [9, 10]. Previously, we reported a patient whose disease course suggested that TPO is involved in thrombocytopenia in AN with severe liver dysfunction [11]. She showed prolonged prothrombin time-international normalized ratio (PT-INR), suggesting decreased hepatic synthetic function leading to a reduction of TPO synthesis [12]. This case implied that prolonged PT-INR could predict thrombocytopenia.

We measured TPO levels in another case of AN with severe liver dysfunction to validate the finding from that previously reported case. A retrospective study was also conducted, aiming to support the hypothesis that prolonged PT-INR can predict thrombocytopenia in AN with severe liver dysfunction.

## Methods

### Case study

We measured plasma TPO levels in a patient with AN admitted to the University of Tokyo Hospital using a chemiluminescent enzyme immunoassay (SRL, Inc., Tokyo, Japan). The reference range obtained from 99 healthy employees of SRL, Inc. was 0.40 ± 0.28 fmol/mL (mean ± 2 standard deviations).

### Retrospective study

We reviewed the medical records of patients with AN who presented with severe liver dysfunction, presumably due to starvation, and were admitted to the University of Tokyo Hospital between April 2012 and March 2021. We excluded patients whose liver enzymes were elevated due to clinically presumed refeeding syndrome. Additionally, patients with liver dysfunction unrelated to AN, such as those with viral hepatitis, were excluded. Severe liver dysfunction was defined as a liver enzyme level > 3 × the upper limit of normal (aspartate aminotransferase [AST] > 120 U/L or alanine aminotransferase [ALT] > 135 U/L) [3, 4]. Data on minimum platelet count during admission were collected. We also obtained the maximum PT-INR value measured between the date of the initial presentation of severe liver dysfunction and the subsequent minimum platelet count.

To compare the PT-INR and platelet counts of patients with and without severe liver dysfunction, we identified matched control patients. Among inpatients with AN without severe liver dysfunction and whose age was within 2 years of each case, we selected a matched participant with the closest body mass index (BMI) to the case. BMI was selected as the matching criterion because it represents AN severity [13]. When no matched control was found, we gradually relaxed the age criteria.

### Statistical analyses

The correlation coefficient between the minimum platelet count and maximum PT-INR was calculated. Furthermore, because TPO affects white blood cell (WBC) production [9], we also quantified the correlation between the minimum WBC count and maximum PT-INR to support the involvement of TPO in thrombocytopenia.

Maximum PT-INR, minimum platelet count, and minimum WBC count were compared between patients with severe liver dysfunction and their matched control. We applied *t*-tests (Student’s or Welch’s) or the Mann–Whitney U-test after examining variance homogeneity using the F-test and normality using the Kolmogorov–Smirnov test. Multivariate linear regressions were performed to adjust for covariates.

Statistical analyses were performed using R version 4.2.2 (R Core Team 2022). Statistical significance was set at *P* < 0.05.

## Results

### Case study

The patient was an 18-year-old Japanese woman with AN without any relevant medical history. Her weight loss started when she was 17 years. She was admitted to a hospital due to loss of consciousness.

**Fig. 1:**
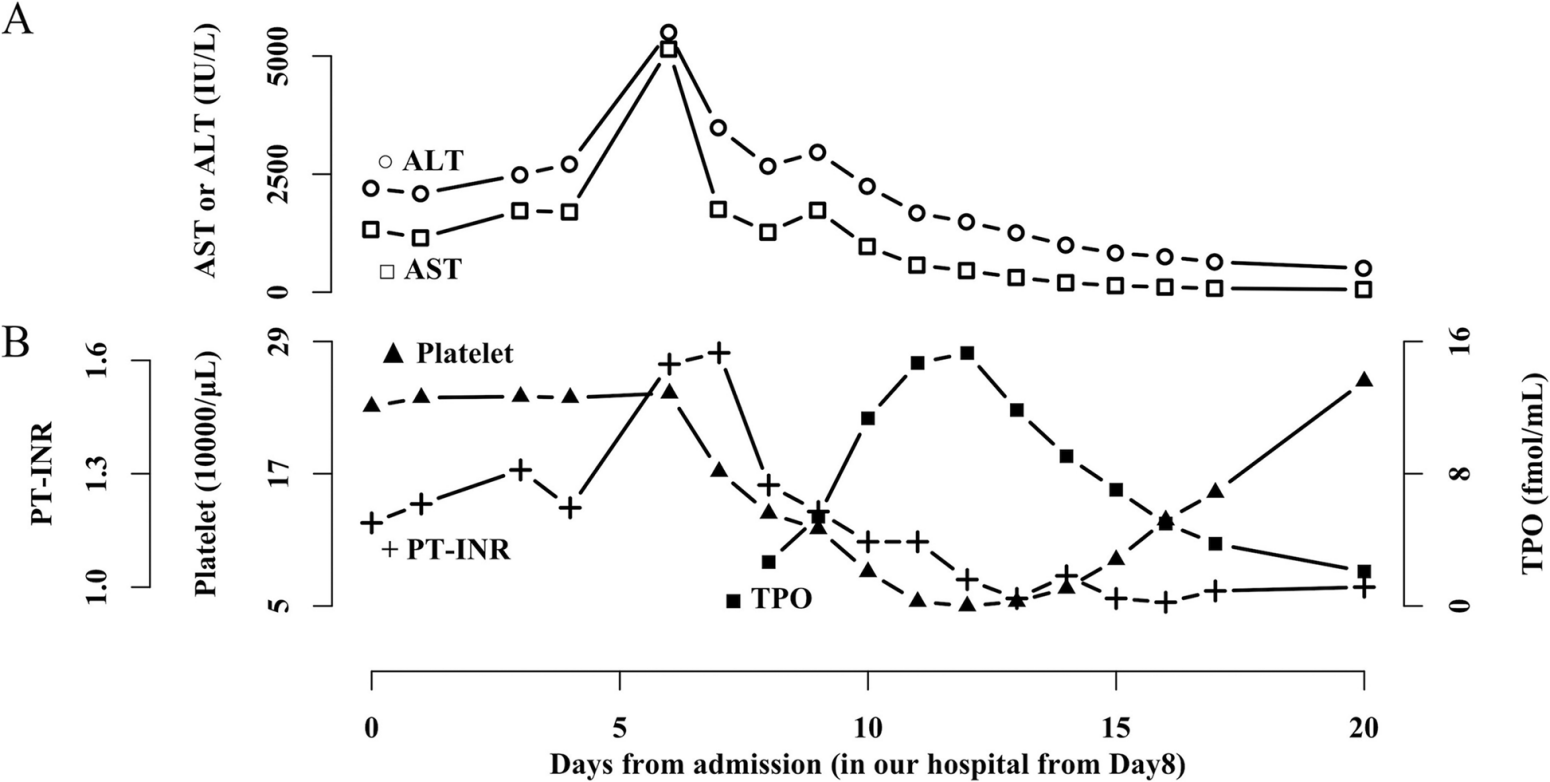
Laboratory data trends. **A** Liver enzyme levels reached their maximum on day 6 and declined afterward. **B** After transfer to our hospital on day 8, the TPO level was first measured. TPO levels increased after improvement in liver enzymes and PT-INR, followed by platelet count recovery. AST, aspartate aminotransferase; ALT, alanine aminotransferase; PT-INR, prothrombin time-international normalized ratio; TPO, thrombopoietin

Figure 1 shows the patient’s laboratory test data obtained after admission. The patient was administered a 300–600 kcal/day diet at the hospital, but her condition worsened, with an AST level of 5145 U/L and an ALT level of 5500 U/L on day 6, and a PT-INR of 1.62 on day 7. Liver dysfunction, until liver enzymes reached their peak, was presumably caused by starvation. A fluid infusion was started on day 6, and the liver enzyme levels started to improve on day 7.

The patient was transferred to the University of Tokyo Hospital on day 8. Her weight was 28.6 kg (BMI, 10.8 kg/m^2^). The TPO level was first measured after transfer. Nutritional therapy (1000 kcal/day) was started through peripheral intravenous infusion and a nasogastric tube and was gradually increased up to 1500 kcal/day on day 12. We found gradual improvements in liver enzyme levels (AST 56 U/L and ALT 505 U/L on day 20) and PT-INR (0.97 on day 13), followed by increased TPO levels (2.66 on day 8 to 15.30 fmol/mL on day 12). Platelet count decreased to 5.0 × 10^4^/μL on day 12 and improved to the normal range thereafter.

### Association between PT-INR and platelet count in the retrospective study

**Fig. 2:**
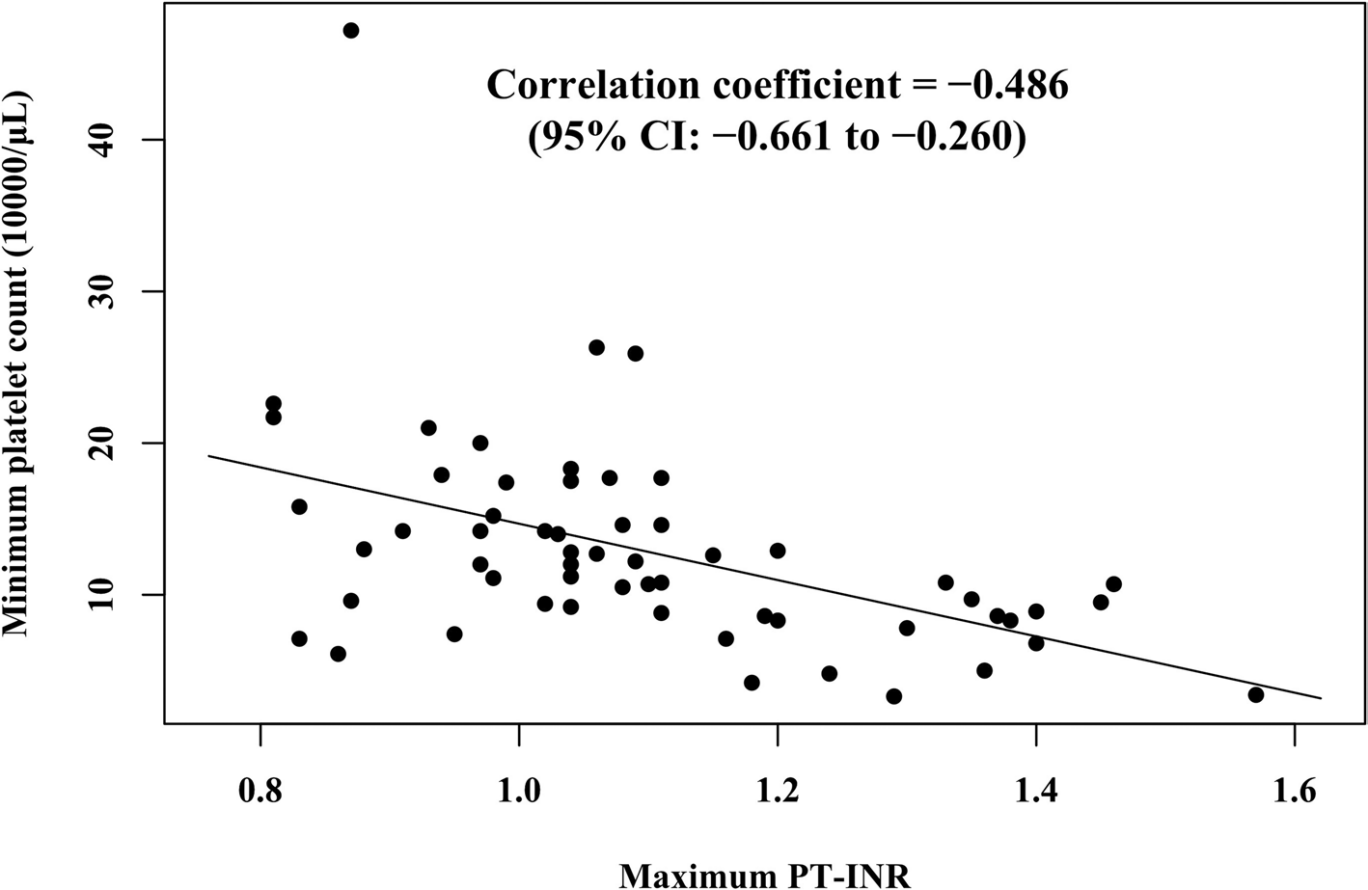
Scatter plot for the association between minimum platelet count and maximum PT-INR in patients with anorexia nervosa presenting with severe liver dysfunction. Measurement of maximum PT-INR temporally preceded that of minimum platelet count. These values were correlated significantly with a coefficient of -0.486. The line indicates the univariate linear regression using the maximum PT-INR as a variable and the minimum platelet count as the outcome. PT-INR, prothrombin time-international normalized ratio

The cohort study included 58 patients (Table 1). The maximum PT-INR and the minimum platelet count were significantly correlated (Fig. 2; correlation coefficient, -0.486; 95% confidence interval [CI], -0.661 to -0.260; *P* < 0.001). The correlation between the maximum PT-INR and the minimum WBC count was also significant (correlation coefficient, -0.407; 95% CI, -0.602 to -0.166; *P* = 0.002).

**Table 1:** Patient characteristics

	Patients with severe liver dysfunction (*N* = 58)	Matched control patients (*N* = 58)	*P*-value
Age (years), mean (SD)	29.9 (14.3)	30.4 (13.7)	0.87^a^
Diagnosis			
ANR, N (%)	43 (74)	41 (71)	N/A
ANBP, N (%)	15 (26)	17 (29)	
Sex			
Male, N (%)	0 (0)	0 (0)	N/A
Female, N (%)	58 (100)	58 (100)	
BMI on admission (kg/m^2^), mean (SD)	11.6 (1.6)	12.3 (1.3)	0.009^a^
Minimum platelet count (10^4^/μL), mean (SD)	12.9 (7.0)	19.6 (4.2)	< 0.001^b^
Minimum WBC count (10^3^/μL), mean (SD)	2.5 (1.4)	2.8 (0.8)	< 0.001^c^
Maximum PT-INR, mean (SD)	1.10 (0.18)	1.00 (0.09)	< 0.001^b^
Maximum AST (U/L), mean (SD)	553 (984)	43 (21)	N/A
Maximum ALT (U/L), mean (SD)	546 (706)	53 (32)	N/A

*Abbreviations*: *ANR* anorexia nervosa restricting type, *ANBP* anorexia nervosa binge-purging type, *BMI* body mass index, *WBC* white blood cell, *PT-INR* prothrombin time-international normalized ratio, *AST* aspartate aminotransferase, *ALT* alanine aminotransferase, *SD* standard deviation, *NA* not available

^a^Student’s *t-*test

^b^Welch’s *t*-test

^c^Mann–Whitney *U*-test

### Comparison with matched control patients

We identified 58 control patients that matched the cases with severe liver dysfunction (Table 1). Although BMI was used as a matching metric, the case group had a lower BMI than the control group. Comparison of these groups showed significant differences in maximum PT-INR, minimum platelet count, and minimum WBC count.

When adjusting for BMI in multivariate linear regression, the case group showed significantly higher PT-INR (β, 0.07; 95% CI, 0.02 to 0.13; *P* = 0.005) and lower platelet count (β, -5.49; 95% CI, -7.47 to -3.52; *P* < 0.001) than control patients.

## Discussion

A patient with AN with severe liver dysfunction showed an improvement in PT-INR after treatment, followed by an increase in TPO level and a further improvement in platelet count. We also conducted a retrospective study, showing a significant correlation between the maximum PT-INR and the minimum platelet count in patients with AN who presented severe liver dysfunction. These patients showed higher PT-INR and lower platelet count than the matched control patients.

The temporal changes in the laboratory test data of the patient were similar to those we reported previously [11]. This strengthened the hypothesis that decreased TPO production due to decreased hepatic synthetic function causes thrombocytopenia and that PT-INR is a predictor of thrombocytopenia. However, TPO values were missing when liver enzymes and PT-INR reached their maximum values. Measurement at this point would be warranted.

Notably, this patient’s TPO values were higher than the healthy subjects’ reference range. Serum TPO level is reportedly regulated by binding to c-mpl on platelet mass [14], and healthy subjects usually have a lower TPO level than patients with thrombocytopenia due to hematologic disease [15]. Therefore, thrombocytopenia may explain the higher TPO values in our case. A previous study by Schiødt et al. also showed an increase in the TPO level of patients with acute liver failure [16]; however, Schiødt et al. noted that this increase was not caused by thrombocytopenia because an inverse relationship between serum TPO level and platelet count was not observed in their study. In contrast, our study found an inverse relationship between them and suggested that the modulation of the TPO level by platelet mass may have existed. Further study is required to determine the extent to which changes in platelet count affect the TPO level.

The significant correlation between maximum PT-INR and minimum platelet count may quantitatively support the hypothesis that prolonged PT-INR predicts thrombocytopenia and is an alternative marker of decreased TPO production. The correlation between PT-INR and WBC count further supports the involvement of TPO in thrombocytopenia, given its involvement in leukocyte production [9]. Furthermore, patients with severe liver dysfunction had higher PT-INR and a lower platelet count than the control group, even after adjustment for BMI. This also suggests that liver dysfunction causes thrombocytopenia.

While this study focused on starvation-induced liver dysfunction, liver dysfunction caused by refeeding, which is generally milder than that caused by starvation, is a common complication of AN [1, 17]. Investigating the platelet count in patients undergoing refeeding may provide additional insights. Furthermore, in a previous study, thrombocytopenia in patients with acute liver failure, mostly caused by acetaminophen, was attributable to systemic inflammation [18]. Further research is necessary to verify whether our hypothesis applies to patients without AN.

This study has several limitations. First, the TPO level in one case was measured only after the transfer, and the cohort study lacked TPO values. Second, some patients did not have their blood data measured daily during hospitalization; thus, the actual maximum PT-INR and minimum platelet count values might not have been obtained. Analyses with daily measurements, such as conducting time-series analyses, may aid in validating our hypothesis. Third, the treatment protocol, such as the amount of calorie intake, was not standardized, which could have affected the results. Finally, we were unable to include several factors related to PT-INR or platelet count, such as vitamin K intake [4, 19].

In conclusion, in patients with AN with severe liver dysfunction, prolonged PT-INR may predict thrombocytopenia, which may be mediated by decreased TPO production due to decreased hepatic synthetic function.

## Data Availability

The dataset is available from the corresponding author upon reasonable request.

## Abbreviations

ALT: Alanine aminotransferase
AST: Aspartate aminotransferase
AN: Anorexia nervosa
BMI: Body mass index
CI: Confidence interval
PT-INR: Prothrombin time-international normalized ratio
TPO: Thrombopoietin
WBC: White blood cell
